# Genomic Blocks in *Aethionema arabicum* Support Arabideae as Next Diverging Clade in Brassicaceae

**DOI:** 10.3389/fpls.2020.00719

**Published:** 2020-06-03

**Authors:** Nora Walden, Thu-Phuong Nguyen, Terezie Mandáková, Martin A. Lysak, Michael Eric Schranz

**Affiliations:** ^1^Biosystematics Group, Wageningen University, Wageningen, Netherlands; ^2^Central European Institute of Technology, Faculty of Science, Masaryk University, Brno, Czechia

**Keywords:** *Aethionema*, Brassicaceae, comparative genomics, genomic blocks, synteny, Arabideae

## Abstract

The tribe Aethionemeae is sister to all other crucifers, making it a crucial group for unraveling genome evolution and phylogenetic relationships within the crown group Brassicaceae. In this study, we extend the analysis of Brassicaceae genomic blocks (GBs) to *Aethionema* whereby we identified unique block boundaries shared only with the tribe Arabideae. This was achieved using bioinformatic methods to analyze synteny between the recently updated genome sequence of *Aethionema arabicum* and other high-quality Brassicaceae genome sequences. We show that compared to the largely conserved genomic structure of most non-polyploid Brassicaceae lineages, GBs are highly rearranged in *Aethionema*. Furthermore, we detected similarities between the genomes of *Aethionema* and *Arabis alpina*, in which also a high number of genomic rearrangements compared to those of other Brassicaceae was found. These similarities suggest that tribe Arabideae, a clade showing conflicting phylogenetic position between studies, may have diverged before diversification of the other major lineages, and highlight the potential of synteny information for phylogenetic inference.

## Introduction

The Brassicaceae is an economically important plant family, containing the *Brassica* crops, rapeseed and several ornamental taxa (e.g., *Aubrieta*, *Iberis*). Due to the availability of abundant genomic resources, such as the high-quality reference genome for model plant *Arabidopsis thaliana*, the family has become a model system for studying plant trait, genome and chromosomal evolution. The Brassicaceae family diverged from its sister-family, the Cleomaceae, ∼43 mya (million years ago) (Schranz and Mitchell-Olds, 2006; Edger et al., 2018). Divergence of tribe Aethionemeae, sister lineage to all other Brassicaceae, with its single genus *Aethionema* occurred ∼32 mya (Hohmann et al., 2015). The subsequent diversification of the rest of the family, or “crown-group,” started ∼23 mya (Hohmann et al., 2015). The crown-group includes ∼3,900 species in 350 genera, grouped into 51 monophyletic tribes^1^ (BrassiBase; Koch et al., 2018). These tribes are further grouped into either three or five major lineages, termed I–III or A–E (Koch and Al-Shehbaz, 2009; Franzke et al., 2011; Huang et al., 2016; Nikolov et al., 2019).

**FIGURE 1:**
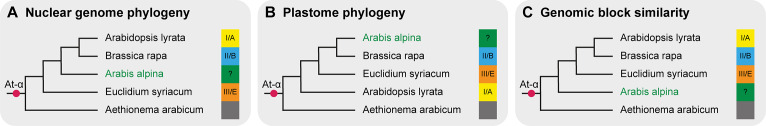
Three potential evolutionary scenarios for the backbone phylogeny of Brassicaceae. **(A)** Nuclear data places lineage III/E as sister to the rest of the family, with Arabideae branching off next and outside of lineage II/B (Kiefer et al., 2019; Nikolov et al., 2019). **(B)** Phylogenetic reconstruction based on plastome data consistently places lineage I/A as sister to lineages II/B and III/E, and Arabideae in lineage II/B or extended lineage II (e.g., Hohmann et al., 2015; Guo et al., 2017). **(C)** Genomic blocks of *Arabis alpina* and *Aethionema arabicum* show some similarities indicating that Arabideae may be the first branching clade in crown group Brassicaceae (this study). The At-α WGD occurred sometime before divergence of Brassicaceae. Note that these three scenarios are not a comprehensive summary of all Brassicaceae phylogenies – other tree topologies have been published (e.g., Beilstein et al., 2010), however, here we only show topologies from recent genomic studies based on a high number of genes. Species names are representatives of their evolutionary lineages and were not necessarily included in the cited studies.

Despite the wealth of sequence information used for recent phylogenetic reconstructions, the deeper nodes of the crown group Brassicaceae, including between lineages, are still not fully resolved. All data show Aethionemeae as the first diverging lineage. However, differing branching orders of the crown-group lineages have been reported. This is largely due to conflicting signals between plastome and nuclear data. Recent phylogenies based on extensive nuclear genome data support lineage III/E, including for example *Euclidium syriacum*, as sister to lineages I/A, including model species *A. thaliana*, and II/B, including the *Brassica* crops (Huang et al., 2016; Kiefer et al., 2019; Nikolov et al., 2019; Figure 1A). Plastome sequence based phylogenies on the other hand consistently place lineage II/B and III/E as sister to lineage I/A (Guo et al., 2017; Mabry et al., 2019; Nikolov et al., 2019; Figure 1B). Additionally, tribe Arabideae, including the important model plant *Arabis alpina*, is placed either as sister to lineages I/A and II/B (Kiefer et al., 2019; Nikolov et al., 2019; Figure 1A) or within lineage II/B (e.g., Hohmann et al., 2015; Guo et al., 2017; Figure 1B). Given the importance of Brassicaceae as a model system, a resolved and reliable backbone phylogeny is a crucial prerequisite for understanding genome and trait evolution on a family-wide scale.

To facilitate comparative genomics and studies of genome evolution, a reference system of genomic blocks (GBs) was established for Brassicaceae genomes (Schranz et al., 2006). Originally inferred from genetic maps and the first available whole genome sequences (*A. thaliana*, *Arabidopsis lyrata*, *Capsella rubella* and *Brassica rapa*), this resulted in the description of the ACK (ancestral crucifer karyotype) with eight chromosomes termed AK1–AK8 and 24 GBs (A–X). Despite its name, the ACK was not the ancestral genome of the Brassicaceae, but rather it can be seen as the hypothetical genome of the MRCA (most recent common ancestor) of lineages I/A and II/B. Since its description, the release of additional whole genome sequences as well as comparative cytogenetic analyses have led to the family-wide expansion of the genomic-block concept and reduction to 22 conserved GBs (Lysak et al., 2016). The PCK (Proto-Calepineae Karyotype, *n* = 7) was described as the ancestral karyotype for clades of lineage II/B (Mandáková and Lysak, 2008), and the CEK (Clade E Karyotype, *n* = 7) as the ancestral karyotype of lineage III/E (Mandáková et al., 2017a). The CEK genome bears some resemblance to the organization of GBs in the *A. alpina* genome (Willing et al., 2015). A high number of within-GB breakpoints compared to the ACK has been observed in both the CEK and *A. alpina* genomes, thus raising the question whether these clades may indeed be more closely related than supported by plastome data. Analysis of GBs can be conducted either with cytogenetic methods, or using bioinformatics. Cytogenetic methods usually rely on Bacterial Artifical Chromosome (BAC) clones, often from *A. thaliana* when studying Brassicaceae species, for chromosome painting. This method has the advantage that even species with little genomic information available can be studied; however, it is limited by the need for BAC clones from more or less closely related taxa. In contrast, bioinformatic methods can be applied even to distantly related species, but high-quality genomic information is required to identify genomic collinearity (syntenic blocks). The evolutionary distance between *Aethionema* and *Arabidopsis* limits the success of chromosome painting, and a high-quality reference genome was so far unavailable. Thus, no species from the more distantly related tribe Aethionemeae has been analyzed in the context of the GBs using either method.

The genus *Aethionema* comprises 57 species (BrassiBase, see footnote 1; Koch et al., 2018). It most likely originated in the Anatolian Diagonal, and dispersed throughout the Irano-Turanian region and large parts of the Mediterranean (Mohammadin et al., 2017). Over the past few years, the genus has been used to study fruit dimorphism (Lenser et al., 2016, 2018; Wilhelmsson et al., 2019) and seed germination (Mérai et al., 2019), in particular using the species *Aethionema arabicum* (e.g., the ERA-CAPS SeedAdapt project). Its divergence occurred sometime after the Brassicaceae-specific At-α WGD (whole genome duplication) (Figure 1). Ancient WGDs are thought to be associated with diversification (Tank et al., 2015), and duplicated genes originating from these events may play an important role for evolving new traits (Hofberger et al., 2013). Remnants of repeated ancient WGDs are found in all land plants (One Thousand Plant Transcriptomes Initiative, 2019), with some of them shared between orders and families, and some family-specific. In addition to At-α, the At-β event specific to the core Brassicales (Edger et al., 2015) is of particular importance to the Brassicaceae. The evolution of glucosinolates, secondary compounds involved in herbivore defense, and the family’s coevolution with Pieridae butterflies is most likely associated with gene family expansion due to WGD following At-β and At-α (Hofberger et al., 2013; Edger et al., 2015). Following polyploidization, genomes often undergo genome size reduction eventually leading to diploidization, a process also referred to as genome fractionation. Genome downsizing is generally accompanied by chromosomal rearrangements and gene loss. The phylogenetic position of *Aethionema* as sister to all other Brassicaceae makes this lineage a crucial link that is needed to understand genome evolution after WGD in Brassicaceae.

The observation that diversifications after WGDs often occur after a considerable time lag and exclude a species-poor sister lineage that shares the WGD has been formalized in the “WGD radiation lag-time model” (Schranz et al., 2012). Diversification of the species-rich and successfully diversifying Brassicaceae crown group contrasted by species-poor tribe Aethionemeae follows this pattern (Schranz et al., 2012). Fractionation and unequal gene loss may be responsible, but this hypothesis still needs to be tested (Schranz et al., 2012). An updated and substantially improved reference genome for *Ae. arabicum* was recently released (*Ae. arabicum* genome v3.0; Nguyen et al., 2019). Its chromosome-level assembly of the eleven linkage groups corresponding to the chromosomes (*n* = 11) allows us to apply the concept of GBs to *Aethionema*, and analyze the genomic structure of the sister clade to all other Brassicaceae in more detail.

Phylogenetics have so far failed to resolve the deeper nodes within Brassicaceae, even using ever larger transcriptome data sets. Instead of relying on nucleotide sequences, using genomic features such as synteny and/or chromosomal rearrangements could therefore prove to be a useful tool to resolve such problematic nodes and disentangle phylogenetic placement of Brassicaceae lineages. Here, we present the syntenic blocks in the genome of *Ae. arabicum* and explore open questions concerning genome evolution and phylogenetics in Brassicaceae: Given the early divergence of *Aethionema* and its position as the species-poor sister group, is its genomic structure similar to the ACK and the largely conserved genomic structure of crown group Brassicaceae? Are the same breakpoints observed between *Aethionema*, *Arabis* and CEK genomes, and can synteny be used to obtain evidence for the phylogenetic position of early diverging lineages? We show that compared to the ACK, the syntenic blocks in the genome of *Aethionema* are broken into a high number of sub-blocks across its linkage groups. Among the high number of breakpoints, we observed, some are shared with *A. alpina* and *E. syriacum* representing the ancestral CEK genome. Our results suggest that Arabideae may have diverged before diversification of lineages I–III.

## Results

### Genomic Blocks in the *Aethionema arabicum* Reference Genome

**FIGURE 2:**
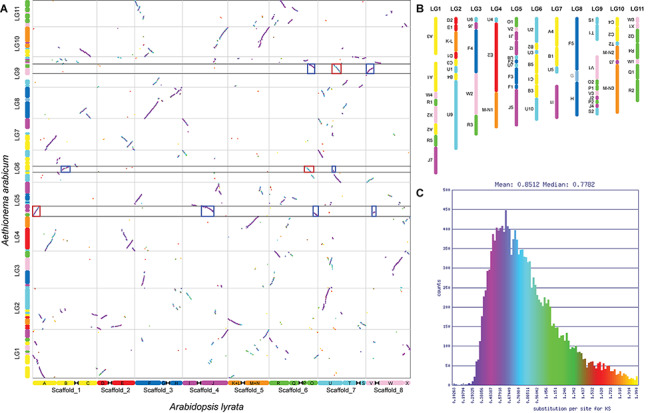
*Aethionema arabicum* genomic blocks. **(A)** Syntenic dotplot of *Ae. arabicum* and *Arabidopsis lyrata*, which closely resembles the ACK. The dotplot was generated using SynMap implemented in CoGe. Syntenic genes are colored by K_s_ values to help differentiate between orthologs and At-α, At-β or segmental duplication derived paralogs. Assignment to genomic blocks is given on the left for *Aethionema* and below for *Arabidopsis*. Red boxes highlight At-α derived blocks syntenic to contiguous blocks detected in the *Aethionema*/*Arabis* comparison, blue boxes their orthologous counterparts. **(B)** Genomic blocks on the eleven linkage groups of *Ae. arabicum*. Up to eight sub-blocks were detected and blocks are ordered relative to the ACK. Inversions are indicated by upside-down block names. For reasons of consistency, genomic block G is shown despite its size below our chosen 20 genes threshold. **(C)** Histogram of synonymous substitution rate K_s_. Color scheme corresponds to that used in panel **(A)**.

Our analysis revealed 13,719 syntenic genes between *Aethionema* and *A. thaliana* that are in syntenic blocks. These blocks are defined as regions sharing at least 20 collinear genes (our chosen threshold for the detection of GBs) when disregarding syntenic regions originating from the At-α, At-β, and segmental duplication events. The duplicated regions could easily be identified using K_s_ values; orthologous blocks generally had median K_s_ values of around 0.77 (purple colored in Figure 2A), while mean K_s_ values for blocks derived from At-α and older duplication events (WGD-derived paralogs) were higher, around 1.37 (blue and turquoise colored in Figure 2A). The average length of the syntenic blocks was 199 ± 183 (mean ± SD) syntenic genes, ranging from 23 to 833 genes. Using the same analysis and parameters on the *Arabis* genome resulted in the detection of 16,588 syntenic genes with an average block length of 313 ± 373 genes, ranging from 25 to 1,507 genes in a block. Here, K_s_ was lower for orthologous blocks (0.41) and At-α derived blocks (1.01). This difference is most likely the result of the additive effect of lineage-specific substitution, leading to a higher number of substitutions in the pairwise comparison of the more divergent species. The difference in syntenic block length is also reflected by the number of syntenic blocks: 69 were detected in the eleven linkage groups of the *Aethionema* genome (Figure 2B), compared to 53 in *Arabis*. All 22 GBs from the ACK (following the updated definition by Lysak et al. (2016) were present in *Aethionema*. However, genomic block G was not detected when setting the minimum number of genes in a syntenic block to 20. When not restricting block size, G was detected (with six syntenic genes) on LG9 between F4 and H, the same position this block has in the ACK.

### Placement of At-α Relative to the Evolution of Brassicaceae

Analysis of the syntenic regions in the genome of *Ae. arabicum* revealed the duplicated regions originating from gene and ancient whole genome duplications. In reciprocal analyses (self–self comparisons), K_s_ values of around 0.8 are generally indicative of duplicates retained from At-α (Schranz et al., 2012). Higher K_s_ values are characteristic for duplicated genes originating from the At-β and older WGDs (Tang and Lyons, 2012). In pairwise analyses, like we conducted here, these characteristic K_s_ values are increased relative to the divergence of the selected species, as each lineage accumulates their own mutations after divergence, thus median K_s_ for orthologs was between 0.58 and 0.94 (Figures 2A,C).

Notably, in K_s_ histograms of *Aethionema* vs. other Brassicaceae the peak from orthologs is almost indistinguishable from that resulting from At-α duplicates. However, the origin of syntenic regions can clearly be distinguished in the syntenic dotplots (Figure 2A and Supplementary Figures 1–3). Median K_s_ in all histograms of pairwise comparisons involving *Aethionema* is around 0.8 (Supplementary Figure 4). This is not the case when comparing other Brassicaceae species, where K_s_ values are between 0.44 and 0.52. The similar K_s_ values between orthologs and paralogs (At-α derived) in *Aethionema* are consistent with only a relatively short time passing between At-α and divergence of *Aethionema* from the rest of Brassicaceae, but diversification of the crown group having occurred with some delay. Nevertheless, the small differences between orthologs and paralogs in *Aethionema* are sufficient to differentiate between the two in the syntenic dotplots (Figure 2A), as well as using median K_s_ of syntenic blocks. In addition, gene content between orthologs is more similar compared to paralogs, due to unequal fractionation, with paralogs generally containing more syntenic genes.

### Conserved Blocks and Boundaries Within Brassicaceae

Most GBs in crown group diploid Brassicaceae species are conserved, i.e., they are not broken into sub-blocks. Interestingly, when within-block breaks and rearrangements are observed, this most often involved AK6 and AK8 [PCK; (Mandáková and Lysak, 2008)] and additionally AK4 [Arabideae; (Mandáková et al., 2020)], with a maximum number of three sub-blocks. This is, however, not the case in *Aethionema*. GBs from all eight chromosomes of the ACK show multiple within-block breakpoints, with the exception of three GBs (G, H, and K–L). The three GBs that did not break into sub-blocks in *Aethionema* are also conserved as a unit throughout most Brassicaceae lineages. Both G and H are present as a single block each on AK3 of the ACK, and they are also conserved in the PCK, CEK, *Arabis* and in the more rearranged *A. thaliana* genome. K-L is conserved as a unit in most lineages of Brassicaceae, but split into K and L in *A. thaliana*, and bioinformatic analysis also detected a small segment (35 genes) of K-L on *A. alpina* chromosome 3, while the largest part of this GB was detected on chromosome 5.

Apart from conserved blocks, also conserved shared GB associations can be observed across the family. While in crown group species only few new GB associations were created through rearrangements (e.g., translocations, inversions), this is the case for almost all blocks in *Aethionema*. Only four GB associations are shared between ACK, PCK, CEK, Arabideae, and *Aethionema*. The A-B association on AK1 can be found on LG-7 (A4-B1), the F-G association on AK3 on LG-8 (F4-G), the G–H association on AK3 is located on LG-8 as well, and the I–J association on AK4 is located on LG-5 (I2–J1). Notably, the I–J association is not found in *Arabis*, but a recent analysis of Arabideae revealed that this association is conserved in *Pseudoturritis turrita*, the sister to all other Arabideae, while it is not retained in later diverging Arabideae genera (Mandáková et al., 2020) and therefore the lack of this association can be considered the derived state in *Arabis*.

### Shared Sub-Block Associations Between Aethionema and Arabis

To explore whether genomic features could help resolve the deeper nodes of the Brassicaceae phylogeny, we searched for shared breakpoints and boundaries between blocks and sub-blocks in the genome sequences of *Aethionema* and *Arabis* that are not present in the ACK. As no chromosome-level assembly from any species of lineage III/E are yet available, we could not extend our analysis to this lineage. Instead, we searched the *E. syriacum* genome for the syntenic regions of interest identified in the *Aethionema*–*Arabis* analysis. Three regions of interest were identified that represent shared block and sub-block boundaries or similar breakpoints.

**FIGURE 3:**
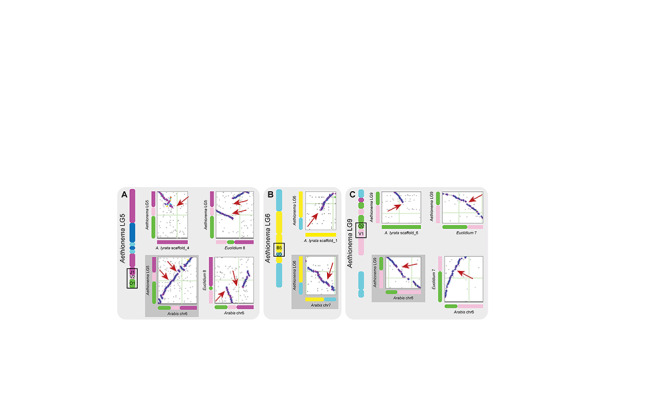
Three syntenic regions in *Aethionema* and *Arabis*. **(A)** Sub-blocks O1, V2 and J1 are present as a unit on LG5 of *Aethionema* and chromosome 6 of *Arabis*, but not contiguous in *A. lyrata*. In *Euclidium*, large parts of O and parts of V and J are missing, and the O-V fragment is inverted relative to *Aethionema* and *Arabis*. **(B)** U3 and B5 are contiguous in *Aethionema* on LG6 and *Arabis* on chromosome 7, but not in *A. lyrata*. **(C)** V1 and O2 on LG9 of *Aethionema* and chromosome 6 of *Arabis* are associated, which is also detected in *Euclidium*, but not in *Arabidopsis*. More detailed figures of the three genomic regions in all pairwise comparisons are shown in Supplementary Figures 5–7.

We identified three shared unique boundaries between *Aethionema* and *Arabis*: J1-V2-O; U3-B5; and V1-O2-P. First, the association J1-V2-O1 from LG-5 (Figure 3A) corresponds to the Ja-V-O association on chromosome Ar6 across Arabideae (Willing et al., 2015; Mandáková et al., 2020). In *Euclidium*, the genome segment containing the J-V-O segment is assembled in one fragment, but contains only partial and inverted V and O orthologs. Comparative chromosome painting did not detect V on chromosome Es3 in *E. syriacum* (Mandáková et al., 2017a). Interestingly, on chromosome Ct6 in *Chorispora tenella* (also lineage III/E), V and a small fragment of neighboring block W have inserted between Ja and Oa (Mandáková et al., 2017a). However, the genome sequence of the species would be required to determine if the breakpoints are identical with those identified in *Aethionema* and Arabideae genomes. Second, the boundary U3-B5 on LG-6 (Figure 3B) is found on chromosome 7 of the *Arabis* genome. The B segment was not detected using chromosome painting on this chromosome in *Arabis* (Willing et al., 2015; Mandáková et al., 2020), but could be detected using SynMap (Supplementary Figure 1A). In *Euclidium*, this fragment of the genome was not assembled contiguously, therefore no conclusions on lineage III/E can be drawn until a chromosome-level assembly becomes available. Finally, the association V1-O2-P1 on LG-9 (Figure 3C) is also detected on chromosome 6 in the *Arabis* genome using bioinformatic methods. In contrast to the other three regions, this association is also shared with *Euclidium* according to our analysis of its genome sequence. However, cytogenetic analyses did not show evidence of block V in vicinity of O in the *Euclidium* genome (Mandáková et al., 2017a), potentially due to the small size of the respective blocks (in *Arabis*, the V sub-block was only 73 syntenic genes long). Detailed figures of the three genomic regions in all pairwise comparisons are shown in Supplementary Figures 5–7.

In support of the potential “ancestral state” of the three aforementioned shared breakpoints between *Aethionema* and *Arabis*, the older At-α derived paralogous blocks are highly syntenic to all three regions (highlighted in red boxes in Figure 2A and Supplementary Figures 1–3; the respective orthologs are highlighted in blue). The J1-V2-O1 block detected in *Aethionema* and *Arabis* is syntenic to a part of A on the AK1, the U3-B5 block is syntenic to a part of O on AK6, and the V1-O2-P1 block from *Aethionema*, *Arabis* and *Euclidium* is syntenic to a part of U on AK7. This similarity of At-α blocks and continuous blocks in *Aethionema* and *Arabis* can be seen as strong evidence for the ancestral status of these genomic regions in the two species, with subsequent rearrangements leading to the blocks building up the ACK.

In genomes of lineage III/E and *Arabis*, blocks from AK4, AK6, AK7, and AK8 are subject to extensive rearrangements and within-block breaks. The association of GBs P and V in particular is observed in *Arabis* on chromosome Ar6 and conserved across Arabideae (Mandáková et al., 2020). Interestingly, the association V3-P2 on LG-9 is also detected in *Aethionema*. However, different block borders are associated, suggesting multiple independent origins. In *Euclidium*, this genomic region was again not assembled contiguously, and no conclusion on lineage III/E can be drawn from genome sequence data. However, cytogenetic analyses have not shown evidence for an association of blocks V and P in any species from this lineage (Mandáková et al., 2017a).

## Discussion

Here, we analyzed GBs in the genome of *Ae arabicum*; comparison with *A. alpina* and *E. syriacum* provide evidence for a new placement of *Arabis* within the Brassicaceae. The phylogenetic position of *Aethionema* as sister of all other Brassicaceae lineages makes this genus particularly interesting in the context of crucifer genome evolution. Due to the earlier availability of genomes and genetic maps of species from lineages I/A and II/B, comparative genomics in Brassicaceae was traditionally conducted relative to the ACK (*n* = 8). The recent update and improvement of the *Ae arabicum* genome sequence (Nguyen et al., 2019) to chromosome-level assembly of the eleven linkage groups has allowed us to apply the system of GBs previously used for other Brassicaceae lineages to tribe Aethionemeae. Compared to other Brassicaceae species, the GBs of the ACK are broken into multiple sub-blocks in *Aethionema*. Interestingly, the genomes of tribe Arabideae (Mandáková et al., 2020) and lineage III/E (Mandáková et al., 2017a) also contain a higher number of sub-blocks than “diploid” genomes from lineage I/A and II/B. A high number of within-block breaks is also observed in diploidized mesopolyploid genomes (e.g., Lysak et al., 2016; Mandáková et al., 2017b) or meso-neopolyploid ones, such as allohexaploid genomes of *Camelina sativa* (Mandáková et al., 2019) and *B. rapa* (Cheng et al., 2013) from lineage I/A and II/B, respectively. However, mesopolyploidization has not been observed in any tribe belonging to lineage III/E (Mandáková et al., 2017a, b), and the Arabideae have also not undergone a WGD post-dating the At-alpha (Willing et al., 2015; Mandáková et al., 2020). Thus, there seem to be two different reasons for elevated fractionation of GBs. While homeologous and ectopic recombination between the duplicated GBs, accompanying post-polyploid diploidization of the mesopolyploid genomes, explains the high number of within-block breakpoints in these genomes, the very definition of ACK and 22 building blocks (Schranz et al., 2006; Lysak et al., 2016) does not reflect the structure of more ancestral genomes of lineage III/E, Arabideae and *Aethionema*. The ACK represents a diploidized genome derived from a more ancestral paleotetraploid genome most likely resembling that of *Ae. arabicum* and to lesser extent those of Arabideae and lineage III/E tribes. With the caveat that phylogenetic position of some crucifer genera and clades remains unresolved as evident by low support values at deeper nodes (Nikolov et al., 2019) and conflicting topologies between studies (e.g., Beilstein et al., 2010; Huang et al., 2016; Nikolov et al., 2019), the phylogenetic placement of ACK is revisited as an ancestral genome of lineage I/A and lineage II/B (Mandáková et al., 2017a).

The backbone phylogeny of Brassicaceae has been a subject of debate in recent years, with conflicting signals from plastome and nuclear genome data, and low resolution at deeper nodes despite large data sets. The use of non-nucleotide genomic data, such as patterns of synteny, may thus help in resolving these nodes. Tools to reconstruct phylogenetic trees based on genome rearrangement patterns have been developed recently (Drillon et al., 2020; Zhao et al., 2020), and first angiosperm-wide phylogenetic reconstructions based on synteny data have shown that these methods may provide alternative phylogenetic evidence for controversial nodes (Zhao et al., 2020). Our analysis of the genome structure of *Aethionema* and *Arabis* in the context of the entire Brassicaceae family was aimed at identifying evidence for the phylogenetic position of Arabideae relative to lineage II/B, where the tribe is consistently placed using plastome (Franzke et al., 2011; Hohmann et al., 2015; Guo et al., 2017; Figure 3A), but not nuclear genome data (Nikolov et al., 2019; Figure 3B), and lineage III/E. Comparative cytogenomic analyses of lineage III/E and Arabideae genera have revealed extensive chromosome reshuffling in these potentially earlier diverging branches of Brassicaceae (Willing et al., 2015; Mandáková et al., 2017a). Our analysis of GBs in *Ae. arabicum* shows that four block associations are shared between *Aethionema* and *Arabis*, indicating that these associations may have been the ancestral state. The breakpoints of these blocks and sub-blocks coincided with assembly borders in the genome sequence of *Euclidium* in one case, and thus we had to rely on evidence from cytogenetic data to infer the status of synteny at this region in lineage III/E. One shared association was observed in *Aethionema*, *Arabis* and *Euclidium*. This boundary notably does not involve any within-block borders, but the outer borders of blocks O and V (from AK 6 and AK7, respectively). Two further associations were shared between *Aethionema* and *Arabis* on LG5 of the *Aethionema* genome. The three sub-blocks (O1-V2-J1) were detected in the same order in *Aethionema* and *Arabis*, but the genome assembly of *Euclidium* indicated two gaps and an inversion at this location. Interestingly, here the V2 sub-block was not located at the edge of GB V from ACK, thus only internal breakpoints were involved.

The presence of identical characters in different lineages can, in short, be explained by two different processes: Either they are derived and originated independently in the respective lineages, or they are ancestral and were lost sometime in the past in the lineages that do not contain them. Two possible explanations and evolutionary scenarios may thus be invoked for the interpretation of our results. The first scenario follows previous interpretations of the ACK as the ancestral genome of Brassicaceae. In this case, the rearranged genomes of lineage III/E, *A. alpina* and *Ae arabicum* are derived from an ancestral Brassicaceae genome similar to the ACK. Their apparent similarity could be the result of frequent reuse of breakpoints. In the second scenario, the blocks from the ACK are the derived state and originated from an ancestral Brassicaceae genome somewhat resembling the genomes of *Aethionema*, *Arabis* and lineage III/E. Having a lower number of required changes, this seems to be the slightly more parsimonious scenario given our current data, and synteny with continuous At-α derived blocks additionally supports our claim. Altogether, our results suggest that the *Arabis* clade diverged first within the Brassicaceae crown group, followed by lineage III/E and finally the most species-rich groups of lineages I/A and II/B (see Figure 1C).

To further advance our understanding of genome evolution in Brassicaceae, genome reconstruction of the family’s most recent common ancestor, the post At-α genome, before divergence of *Aethionema*, is needed. This would allow for a redefinition of GBs relative to this presumed ancestral genome and for analysis of genome evolution in all lineages of the family. Whereas multiple high-quality genomes from lineages I/A and II/B are available, comparable genome sequences are not available yet for other crucifer clades. Chromosome-level assemblies from lineage III/E would allow us to test hypotheses regarding the backbone phylogeny and placement of lineage III/E as well as tribe Arabideae in more detail. In particular, the similarity of lineage III/E genomes with the ACK should be studied further. Additionally, the genome sequences of other *Aethionema* species, preferably some that diverged from *Ae. arabicum* early in the evolution of the tribe, would allow us to determine an ancestral karyotype of tribe Aethionemeae and to conclude whether the eleven *Aethionema* linkage groups represent the relic At-α genome frozen in time or a reshuffled paleotetraploid genome. This would also give us the opportunity to study the genome evolution of this species-poor sister clade, and could shed some light on why Aethionemeae did not diversify like the rest of Brassicaceae.

## Materials and Methods

### Genomic Block Identification

We identified syntenic blocks in the updated reference genome of *Ae. arabicum* v.3 (Nguyen et al., 2019) relative to the 22 GBs in ACK (Schranz et al., 2006; Lysak et al., 2016). Note that throughout the manuscript, we refer to bioinformatically or cytogenomically detected syntenic blocks in extant species as “syntenic blocks” or simply “blocks” and to those of the ACK as “genomic blocks” or “GBs.” The CoGe platform^2^ (Lyons et al., 2008) was used to detect syntenic regions between *Ae. arabicum* and *A. thaliana*, as GBs in the ACK are defined using the *A. thaliana* gene IDs as start and end coordinates. Orthologous genes were identified using the BlastZ algorithm, and synteny was identified using DAGchainer (Haas et al., 2004). To obtain larger syntenic regions, we set the maximum distance between two matches in DAGchainer to 25, and only retained blocks with a minimum number of 20 retained pairs. For reasons of consistency, an exception was made for block G, which could only be identified with default settings, as it only contained six genes. We merged syntenic regions using QuotaAlign (Tang et al., 2011) with a maximum distance of 50 genes. In order to differentiate between syntenic blocks representing the GBs and those retained from At-α, we calculated the synonymous substitution rate (K_s_) using CodeML (Yang, 2007) as implemented in CoGe. For values <2, K_s_ is relatively linear with time (Vanneste et al., 2013) and can be used to distinguish between orthologs between species, At-α derived genes or blocks with a K_s_ value around 0.8 (Schranz et al., 2012), recently duplicated genes, and duplicated genes with an even older origin, for example from the At-β WGD. Blocks with median K_s_ values corresponding to a WGD were discarded. Note that due to the additive effect of lineage-specific substitutions, K_s_ values for pairwise comparisons are higher with longer divergence time. In the *Aethionema*-*Arabidopsis* comparison, orthologs had a mean K_s_ of 0.77, while it was 0.42 in the Arabis-Arabidopsis comparison; mean Ks values of At-α derived paralogs were 1.37 and 1.01, respectively. Additionally, we checked each syntenic block for redundancy, i.e., if a block spanning the same part of the ACK was present more than once; the block with lower K_s_ was retained. The *Aethionema* blocks were generated from the remaining syntenic blocks; in the few cases where neighboring blocks from the same GB were not merged by QuotaAlign because of their distance, we merged them manually. Note that while gene names from *A. thaliana* define the borders, direction of blocks is given relative to the ACK. For visualization, we generated a syntenic dotplot and K_s_ plot using *Ae. arabicum* and *Arabidopsis lyrata*, a species with high similarity to the ACK. In this case, default parameters were set for DAGchainer and syntenic regions were not merged. Minimum length of chromosomes was set to 5,000,000 bp to retain only chromosomes from the genomes.

### Comparison With Other Species

We compared the GBs from Aethionema with those from other species by running similar CoGe analyses with the following three species pairs: *Ae. arabicum* – *A. lyrata* (representative of lineage I/A and close to ACK), *Ae. arabicum* – *A. alpina* (unclear phylogenetic position), *Ae. arabicum* – *E. syriacum* (representative of lineage III/E) and *A. alpina* – *A. thaliana*. Blocks were only reconstructed for *A. alpina* (using the same parameters as above) and used to identify boundaries between (sub-)blocks shared between *Aethionema* and *Arabis*. Syntenic dotplots of these regions were finally compared between all species. Minimum length of chromosomes was again set to 5,000,000 bp to retain only chromosomes from the genomes, except for *Euclidium*, where shorter chromosomal length of 500,000 bp was allowed. The genome sequence of *Euclidium* is not quite assembled on a chromosomal-level, and block boundaries sometimes coincided with assembly borders. As we could not determine whether this was an artefact of assembly or the syntenic block boundary was located at the chromosome (arm) edge, we also used cytogenetic evidence from the literature for interpretation of our results.

## Data Availability Statement

Publicly available datasets were analyzed in this study and download links are given in Supplementary Table 1.

## Author Contributions

MS conceived the study. NW and T-PN analyzed the data. NW wrote the manuscript with input from TM, ML, and MS.

## Conflict of Interest

The authors declare that the research was conducted in the absence of any commercial or financial relationships that could be construed as a potential conflict of interest. The reviewer ZL declared past co-authorship with one of the authors, MS, to the handling editor.

## Footnotes

1 https://brassibase.cos.uni-heidelberg.de/

2 https://genomevolution.org/coge/

## References

[B1] Beilstein M. A. Nagalingum N. S. Clements M. D. Manchester S. R. Mathews S. (2010). Dated molecular phylogenies indicate a miocene origin for *Arabidopsis thaliana*. *Proc. Natl. Acad. Sci. U.S.A.* 107 18724–18728. 10.1073/pnas.0909766107 20921408 PMC2973009

[B2] Cheng F. Mandáková T. Wu J. Xie Q. Lysak M. A. Wang X. (2013). Deciphering the diploid ancestral genome of the mesohexaploid *Brassica rapa*. *Plant Cell* 25 1541–1554. 10.1105/tpc.113.110486 23653472 PMC3694691

[B3] Drillon G. Champeimont R. Oteri F. Fischer G. Carbone A. (2020). Phylogenetic reconstruction based on synteny block and gene adjacencies. *Molecular Biol. Evol.* msaa114. 10.1093/molbev/msaa114 32384156 PMC7475045

[B4] Edger P. P. Hall J. C. Harkess A. Tang M. Coombs J. Mohammadin S. (2018). *Brassicales* phylogeny inferred from 72 plastid genes: a reanalysis of the phylogenetic localization of two paleopolyploid events and origin of novel chemical defenses. *Am. J. Bot.* 105 463–469. 10.1002/ajb2.1040 29574686

[B5] Edger P. P. Heidel-Fischer H. M. Bekaert M. Rota J. Glöckner G. Platts A. E. (2015). The butterfly plant arms-race escalated by gene and genome duplications. *Proc. Natl. Acad. Sci. U.S.A.* 112 8362–8366. 10.1073/pnas.1503926112 26100883 PMC4500235

[B6] Franzke A. Lysak M. A. Al-Shehbaz I. A. Koch M. A. Mummenhoff K. (2011). Cabbage family affairs: the evolutionary history of *Brassicaceae*. *Trends Plant Sci.* 16 108–116. 10.1016/j.tplants.2010.11.005 21177137

[B7] Guo X. Liu J. Hao G. Zhang L. Mao K. Wang X. (2017). Plastome phylogeny and early diversification of *Brassicaceae*. *BMC Genomics* 18:e176 10.1186/s12864-017-3555-3553 PMC5312533 28209119

[B8] Haas B. J. Delcher A. L. Wortman J. R. Salzberg S. L. (2004). DAGchainer: a tool for mining segmental genome duplications and synteny. *Bioinformatics* 20 3643–3646. 10.1093/bioinformatics/bth397 15247098

[B9] Hofberger J. A. Lyons E. Edger P. P. Chris Pires J. Eric Schranz M. (2013). Whole genome and tandem duplicate retention facilitated glucosinolate pathway diversification in the mustard family. *Genome Biol. Evol.* 5 2155–2173. 10.1093/gbe/evt162 24171911 PMC3845643

[B10] Hohmann N. Wolf E. M. Lysak M. A. Koch M. A. (2015). A time-calibrated road map of *Brassicaceae* species radiation and evolutionary history. *Plant Cell* 27 2770–2784. 10.1105/tpc.15.00482 26410304 PMC4682323

[B11] Huang C.-H. Sun R. Hu Y. Zeng L. Zhang N. Cai L. (2016). Resolution of *Brassicaceae* phylogeny using nuclear genes uncovers nested radiations and supports convergent morphological evolution. *Mol. Biol. Evol.* 33 394–412. 10.1093/molbev/msv226 26516094 PMC4866547

[B12] Kiefer C. Willing E.-M. Jiao W.-B. Sun H. Piednoël M. Hümann U. (2019). Interspecies association mapping links reduced CG to TG substitution rates to the loss of gene-body methylation. *Nat. Plants* 5 846–855. 10.1038/s41477-019-0486-489 31358959

[B13] Koch M. A. Al-Shehbaz I. A. (2009). “Molecular systematics and evolution of ‘wild’ crucifers (*Brassicaceae* or *Cruciferae*),” in *Biology and Breeding of Crucifers*, ed. Gupta S. K. (Boca Raton, FL: CRC Press), 1–19.

[B14] Koch M. A. German D. A. Kiefer M. Franzke A. (2018). Database taxonomics as key to modern plant biology. *Trends Plant Sci.* 23 4–6. 10.1016/j.tplants.2017.10.005 29146431

[B15] Lenser T. Graeber K. Cevik O. S. Adiguzel N. Donmez A. A. Grosche C. (2016). Developmental control and plasticity of fruit and seed dimorphism in *Aethionema arabicum*. *Plant Physiol.* 172 1691–1707. 10.1104/pp.16.00838 27702842 PMC5100781

[B16] Lenser T. Tarkowská D. Novák O. Wilhelmsson P. K. I. Bennett T. Rensing S. A. (2018). When the BRANCHED network bears fruit: how carpic dominance causes fruit dimorphism in *Aethionema*. *Plant J.* 94 352–371. 10.1111/tpj.13861 29418033

[B17] Lyons E. Pedersen B. Kane J. Alam M. Ming R. Tang H. (2008). Finding and comparing syntenic regions among Arabidopsis and the outgroups papaya, poplar, and grape: CoGe with Rosids. *Plant Physiol.* 148 1772–1781. 10.1104/pp.108.124867 18952863 PMC2593677

[B18] Lysak M. A. Mandáková T. Schranz M. E. (2016). Comparative paleogenomics of crucifers: ancestral genomic blocks revisited. *Curr. Opin. Plant Biol.* 30 108–115. 10.1016/j.pbi.2016.02.001 26945766

[B19] Mabry M. E. Brose J. M. Blischak P. D. Sutherland B. Dismukes W. T. Bottoms C. A. (2019). Phylogeny and multiple independent whole-genome duplication events in the *Brassicales*. *BioRxiv* [Preprint]. 10.1101/789040 PMC7496422 32830865

[B20] Mandáková T. Hloušková P. German D. A. Lysak M. A. (2017a). Monophyletic origin and evolution of the largest crucifer genomes. *Plant Physiol.* 174 2062–2071. 10.1104/pp.17.00457 28667048 PMC5543974

[B21] Mandáková T. Li Z. Barker M. S. Lysak M. A. (2017b). Diverse genome organization following 13 independent mesopolyploid events in *Brassicaceae* contrasts with convergent patterns of gene retention. *Plant J.* 91 3–21. 10.1111/tpj.13553 28370611

[B22] Mandáková T. Hloušková P. Koch M. A. Lysak M. A. (2020). Genome evolution in Arabideae was marked by frequent centromere repositioning. *Plant Cell* 32 650–665. 10.1105/tpc.19.00557 31919297 PMC7054033

[B23] Mandáková T. Lysak M. A. (2008). Chromosomal phylogeny and karyotype evolution in x = 7 crucifer species (*Brassicaceae*). *Plant Cell* 20 2559–2570. 10.1105/tpc.108.062166 18836039 PMC2590746

[B24] Mandáková T. Pouch M. Brock J. R. Al-Shehbaz I. A. Lysak M. A. (2019). Origin and evolution of diploid and allopolyploid *Camelina* genomes were accompanied by chromosome shattering. *Plant Cell* 31:2596. 10.1105/tpc.19.00366 31451448 PMC6881126

[B25] Mérai Z. Graeber K. Wilhelmsson P. Ullrich K. K. Arshad W. Grosche C. (2019). *Aethionema arabicum*: a novel model plant to study the light control of seed germination. *J. Exp. Bot.* 70 3313–3328. 10.1093/jxb/erz146 30949700 PMC6598081

[B26] Mohammadin S. Peterse K. Kerke S. J. van de Chatrou L. W. Dönmez A. A. Mummenhoff K. (2017). Anatolian origins and diversification of *Aethionema*, the sister lineage of the core Brassicaceae. *Am. J. Bot.* 104 1042–1054. 10.3732/ajb.1700091 28743759

[B27] Nguyen T.-P. Mühlich C. Mohammadin S. van den Bergh E. Platts A. E. Haas F. B. (2019). Genome improvement and genetic map construction for *Aethionema arabicum*, the first divergent branch in the *Brassicaceae* family. *G3* 9:3521. 10.1534/g3.119.400657 31554715 PMC6829135

[B28] Nikolov L. A. Shushkov P. Nevado B. Gan X. Al-Shehbaz I. A. Filatov D. (2019). Resolving the backbone of the *Brassicaceae* phylogeny for investigating trait diversity. *New Phytol.* 222 1638–1651. 10.1111/nph.15732 30735246

[B29] One Thousand Plant Transcriptomes Initiative (2019). One thousand plant transcriptomes and the phylogenomics of green plants. *Nature* 574 679–685. 10.1038/s41586-019-1693-2 31645766 PMC6872490

[B30] Schranz M. Lysak M. Mitchellolds T. (2006). The ABC’s of comparative genomics in the *Brassicaceae*: building blocks of crucifer genomes. *Trends Plant Sci.* 11 535–542. 10.1016/j.tplants.2006.09.002 17029932

[B31] Schranz M. E. Mitchell-Olds T. (2006). Independent ancient polyploidy events in the sister families *Brassicaceae* and *Cleomaceae*. *Plant Cell* 18 1152–1165. 10.1105/tpc.106.041111 16617098 PMC1456871

[B32] Schranz M. E. Mohammadin S. Edger P. P. (2012). Ancient whole genome duplications, novelty and diversification: the WGD radiation lag-time model. *Curr. Opin. Plant Biol.* 15 147–153. 10.1016/j.pbi.2012.03.011 22480429

[B33] Tang H. Lyons E. (2012). Unleashing the genome of brassica rapa. *Front. Plant Sci.* 3:172. 10.3389/fpls.2012.00172 22866056 PMC3408644

[B34] Tang H. Lyons E. Pedersen B. Schnable J. C. Paterson A. H. Freeling M. (2011). Screening synteny blocks in pairwise genome comparisons through integer programming. *BMC Bioinform.* 12:102. 10.1186/1471-2105-12-102 21501495 PMC3088904

[B35] Tank D. C. Eastman J. M. Pennell M. W. Soltis P. S. Soltis D. E. Hinchliff C. E. (2015). Nested radiations and the pulse of angiosperm diversification: increased diversification rates often follow whole genome duplications. *New Phytol.* 207 454–467. 10.1111/nph.13491 26053261

[B36] Vanneste K. Van de Peer Y. Maere S. (2013). Inference of genome duplications from age distributions revisited. *Mol. Biol. Evol.* 30 177–190. 10.1093/molbev/mss214 22936721

[B37] Wilhelmsson P. K. I. Chandler J. O. Fernandez-Pozo N. Graeber K. Ullrich K. K. Arshad W. (2019). Usability of reference-free transcriptome assemblies for detection of differential expression: a case study on *Aethionema arabicum* dimorphic seeds. *BMC Genom.* 20:95 10.1186/s12864-019-5452-5454 PMC6354389 30700268

[B38] Willing E.-M. Rawat V. Mandáková T. Maumus F. James G. V. Nordström K. J. V. (2015). Genome expansion of *Arabis alpina* linked with retrotransposition and reduced symmetric DNA methylation. *Nat. Plants* 1:14023. 10.1038/nplants.2014.23 27246759

[B39] Yang Z. (2007). PAML 4: phylogenetic analysis by maximum likelihood. *Mol. Biol. Evol.* 24 1586–1591. 10.1093/molbev/msm088 17483113

[B40] Zhao T. Xue J. Kao S. Li Z. Zwaenepoel A. Schranz M. E. (2020). Novel phylogeny of angiosperms inferred from whole-genome microsynteny analysis. *bioRxiv* [Preprint]. 10.1101/2020.01.15.908376 PMC8190143 34108452

