# Supplementary material for: Genomic Blocks in Aethionema arabicum Support Arabideae as Next Diverging Clade in Brassicaceae
Source: Front Plant Sci. 2020 Jun 3;11:719. doi: 10.3389/fpls.2020.00719 (PMC7286309; doi:10.3389/fpls.2020.00719)
Supplement: Supplementary file 1 [file Data_Sheet_1.PDF]

Supplementary Material

1.1 Supplementary Tables

Supplementary Table S1. Genomic resources. Species, genome version, reference and download link are given.

	Version	Reference	Download link
Aethionema arabicum	v3.1	Nguyen et al. (2019)	https://genomevolution.org/coge/GenomeInfo.pl?gid=36061
Arabis alpina	MPIPZ.v5	Willing et al. (2015); Jiao et al. (2017)	https://www.ncbi.nlm.nih.gov/assembly/GCA_900128785.1
Euclidium syriacum	MPIPZ.v1	Jiao et al. (2017)	https://www.ncbi.nlm.nih.gov/assembly/GCA_900116095.1/
Arabidopsis lyrata	v1.0	Hu et al. (2011)	https://www.ncbi.nlm.nih.gov/assembly/GCF_000004255.2

1.2 Supplementary Figures

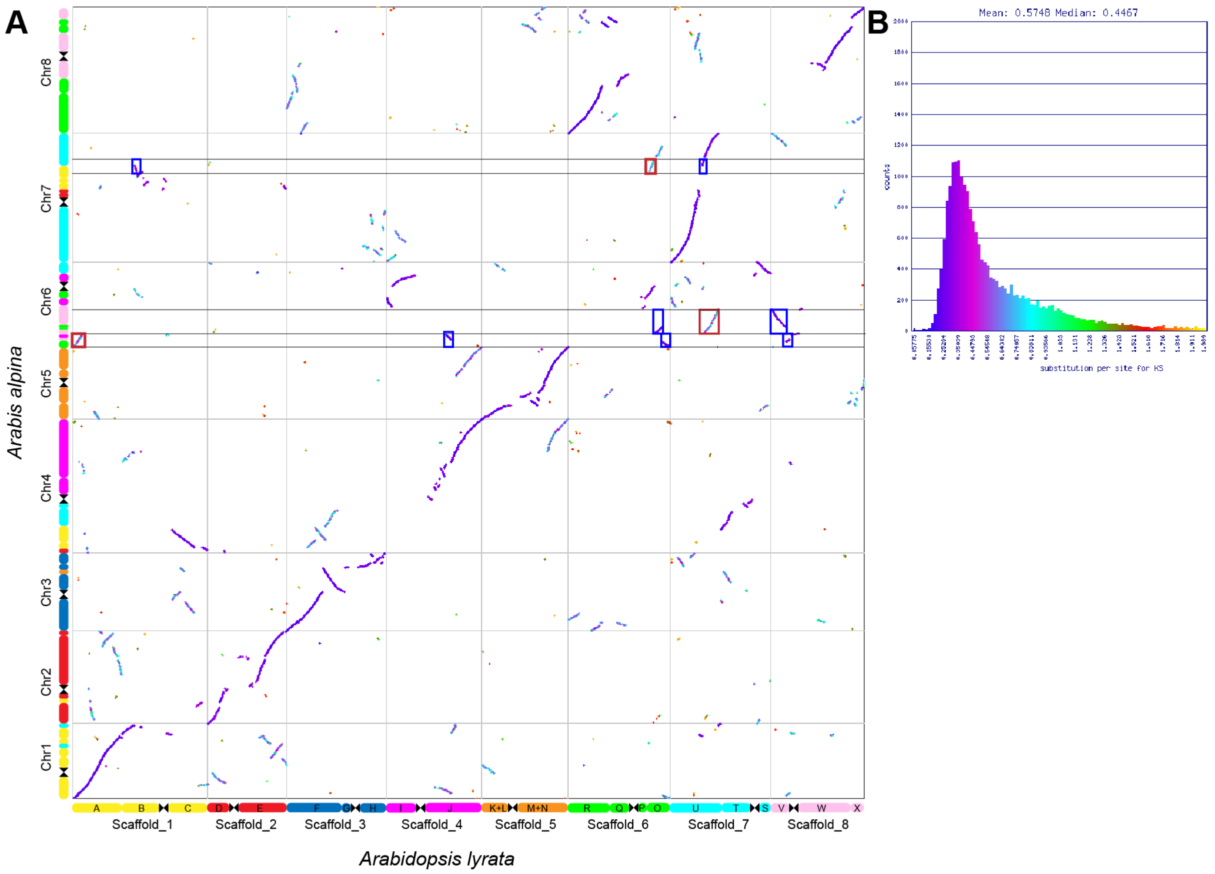

Supplementary Figure 1. Synteny between *Arabidopsis lyrata* and *Arabis alpina.* (A) Syntenic dotplot of *Arabis alpina* and *Arabidopsis lyrata*, which closely resembles the ACK. The dotplot was generated using SynMap implemented in CoGe. Syntenic genes are colored by K_S values to help differentiate between orthologs and At-α or At-β derived paralogs. Assignment to genomic blocks is given on the left for *Arabis* and below for *Arabidopsis*. (B) Histogram of synonymous substitution rate K_S for the syntenic genes between *Arabis alpina* and *Arabidopsis lyrata*. Color scheme corresponds to that used in (A). Red boxes highlight At-α derived blocks syntenic to contiguous blocks detected in the *Aethionema*/*Arabis* comparison (Fig. 3), blue boxes their corresponding orthologs. Compared to the paralogs, orthologs are not continuous across the genomic block borders.

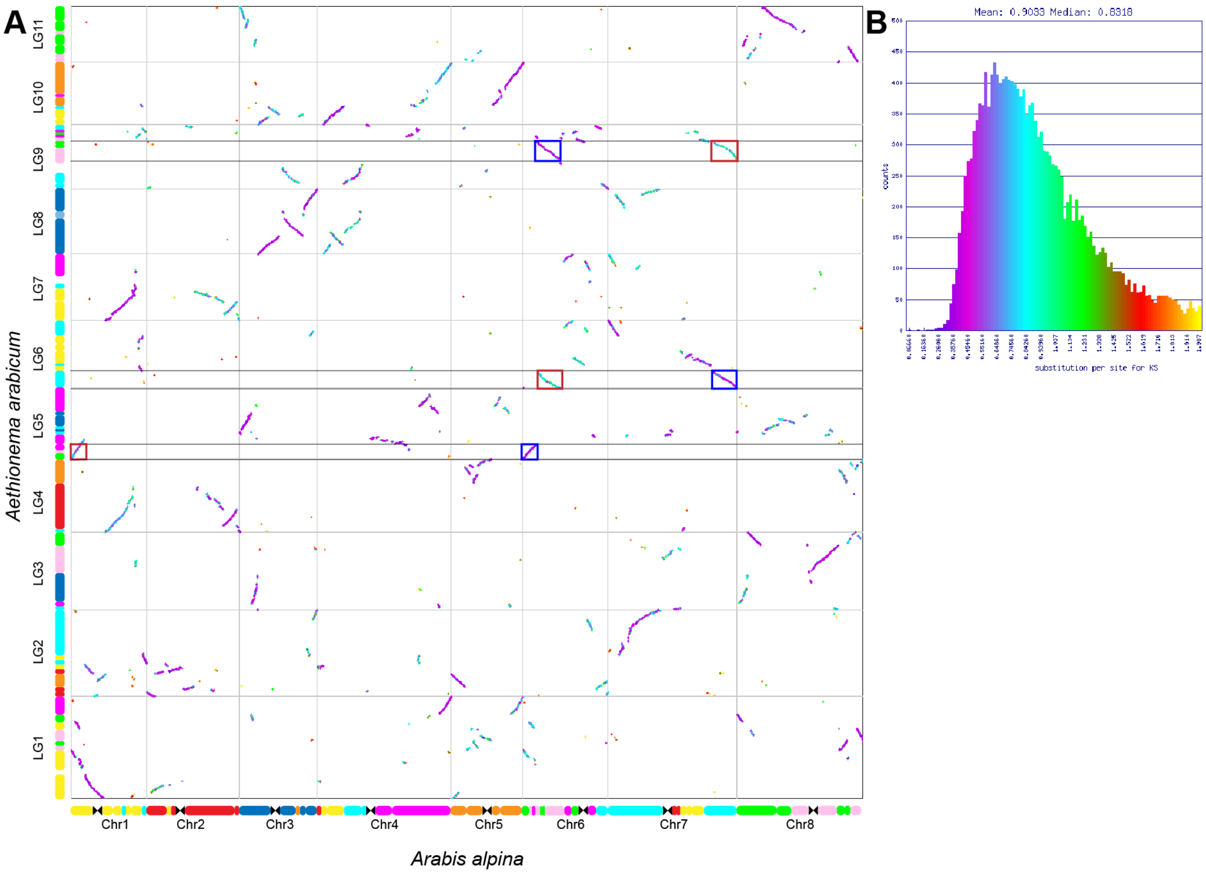

Supplementary Figure 2. Synteny between *Arabis alpina* and *Aethionema arabicum.* (A) Syntenic dotplot of *Aethionema arabicum* and *Arabis alpina*. The dotplot was generated using SynMap implemented in CoGe. Syntenic genes are colored by K_s values to help differentiate between orthologs and At-α or At-β derived paralogs. Assignment to genomic blocks is given on the left for *Aethionema* and below for *Arabis*. (B) Histogram of synonymous substitution rate K_s for the syntenic genes between *Aethionema arabicum* and *Arabis alpina*. Color scheme corresponds to that used in (A). Red boxes highlight At-α derived blocks syntenic to contiguous blocks detected in the *Aethionema*/*Arabis* comparison (Fig. 3), blue boxes their orthologs. Here, the orthologs are continuous across the genomic block borders. Centromere position in *Arabis* follows Mandáková et al. (2020).

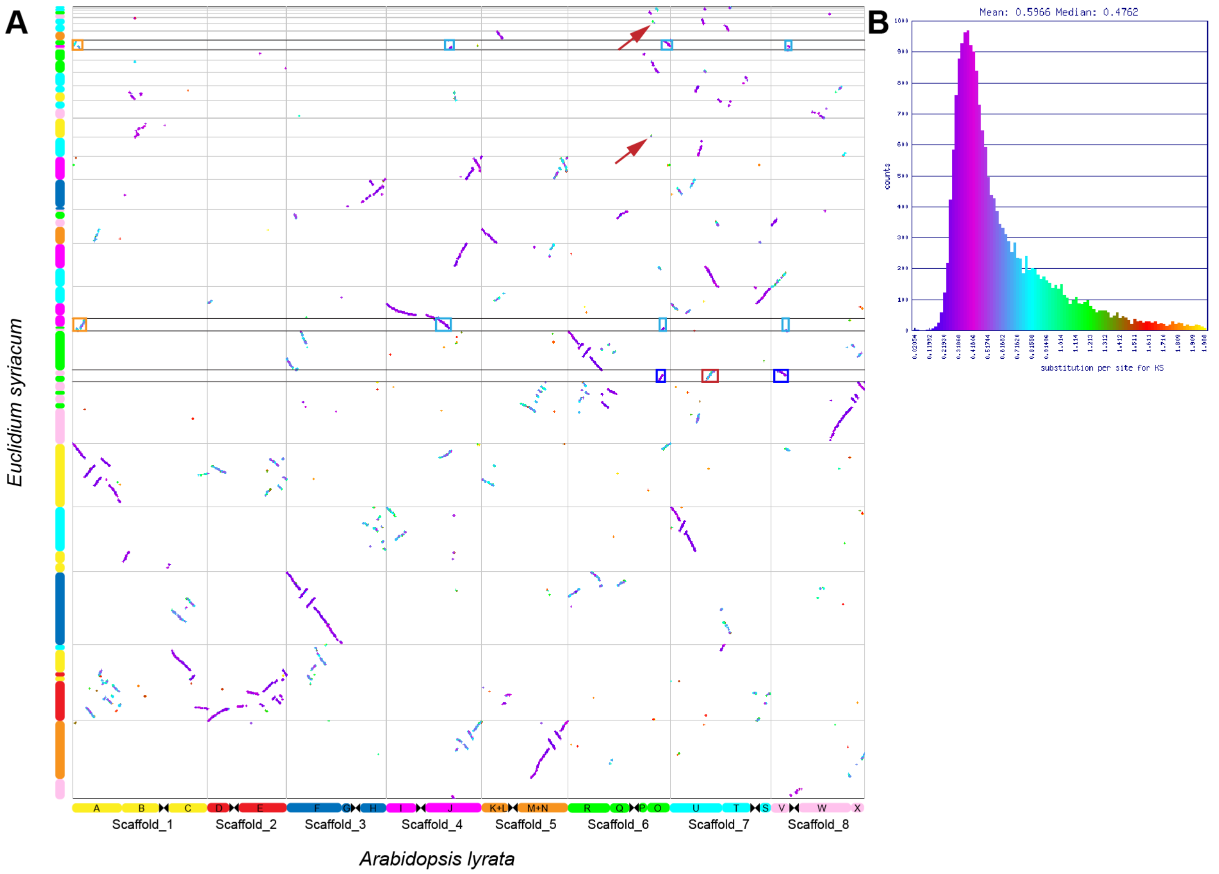

Supplementary Figure 3. Synteny between *Arabidopsis lyrata* and *Euclidium syriacum*. (A) Syntenic dotplot of *Arabidopsis lyrata* and *Euclidium syriacum*. The dotplot was generated using SynMap implemented in CoGe. Syntenic genes are colored by K_S values to help differentiate between orthologs and At-α or At-β derived paralogs. Assignment to genomic blocks is given on the left for *Euclidium* and below for *Arabidopsis*. (B) Histogram of synonymous substitution rate K_S for the syntenic genes between *Arabidopsis lyrata* and *Euclidium syriacum*. Color scheme corresponds to that used in (A). Red boxes highlight At-α derived blocks syntenic to contiguous blocks detected in the *Aethionema*/*Arabis* comparison (Fig. 3), blue boxes their orthologs. Due to the assembly quality of the *Euclidium* genome, the ortholog corresponding to the At-α block on scaffold_1 of *Arabidopsis* seems not assembled in continuously, but on two scaffolds in *Euclidium* (orange and light blue boxes). Furthermore, only two very short fragments corresponding to the At-α block on scaffold_6 of *Arabidopsis* were detected at the ends of two shorter scaffolds in *Euclidium* (red arrows).

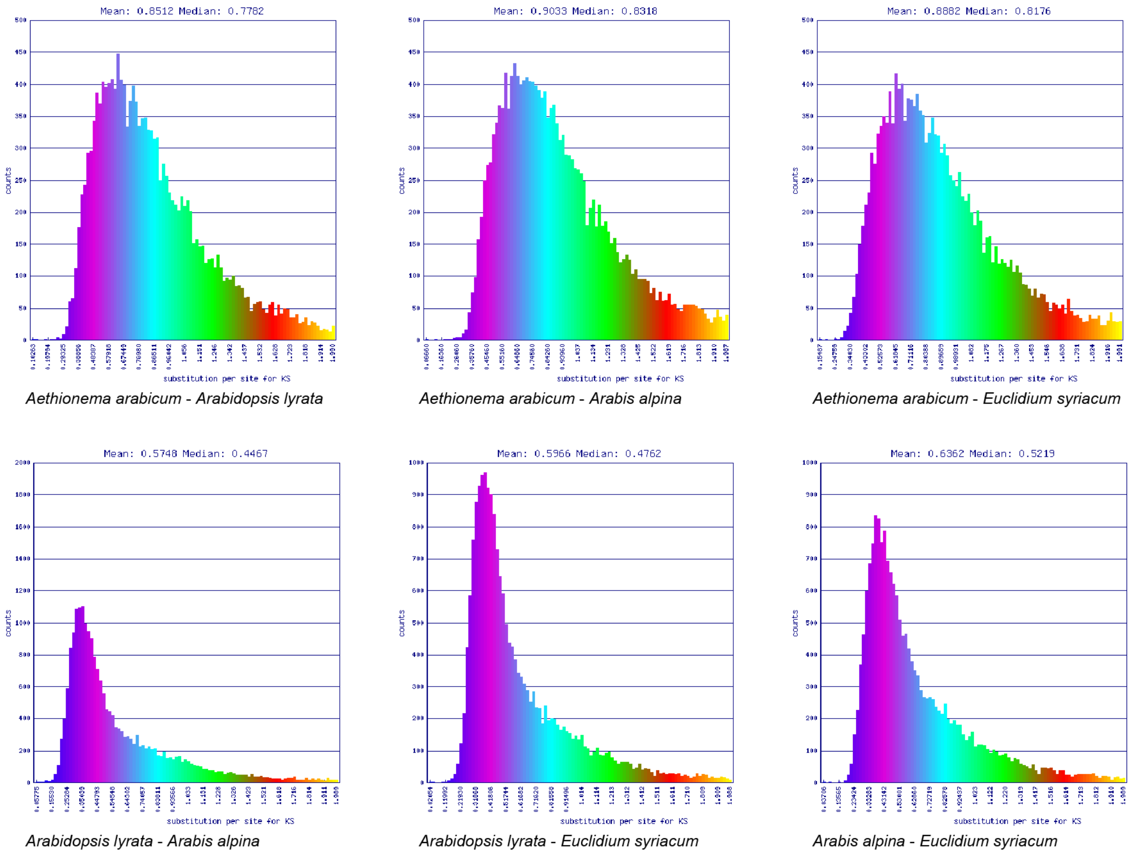

Supplementary Figure 4. Pairwise synonymous substitution rates. Histograms of all pairwise comparisons for *Aethionema arabicum, Arabidopsis lyrata, Arabis alpina* and *Euclidium syriacum* are shown with a maximum K_s value of 2. Colors correspond to those shown in the syntenic dotplots in Fig. 2 and Supplementary Figure 1. Median K_s for all comparisons including *Aethionema* was ~0.8, and between 0.44 and 0.52 for more closely related species, consistent with an older divergence time of *Aethionema* from the rest, close to the time of the At-α WGD.

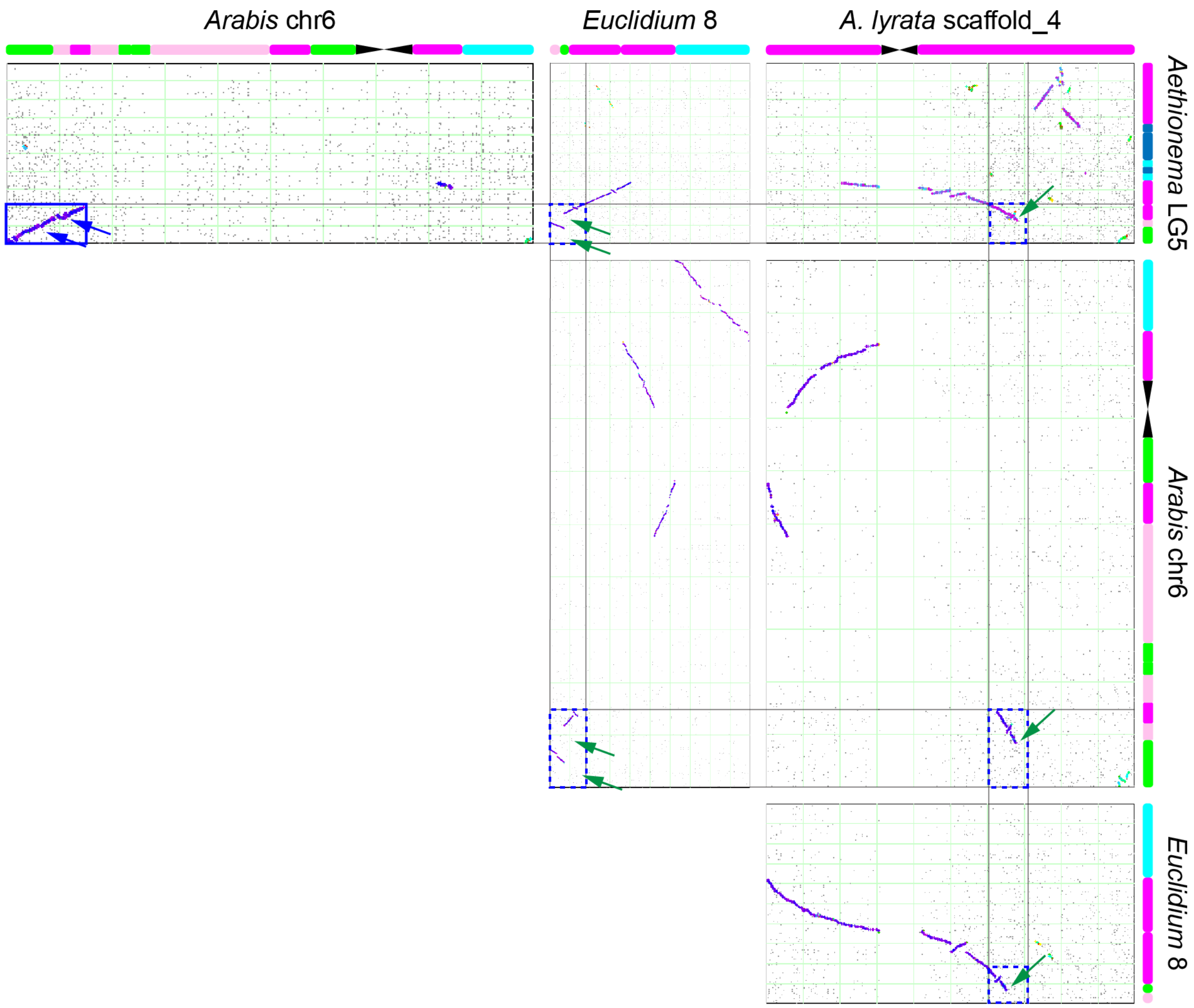

Supplementary Figure 5. Genomic block continuity from Fig. 3A. A detailed, chromosomal view of the break points shown in Fig. 3A in pairwise comparisons between *Aethionema*, *Arabis*, *Euclidium* and *Arabidopsis*. The genomic region continuous in the pairwise syntenic dotplot of *Aethionema* and *Arabis*, spanning the blocks O1-V2-J1 in *Aethionema*, is highlighted in blue. Non-continuous orthologs of this region in the other pairwise comparisons are highlighted with a blue dashed line. Green arrows indicate the break points, blue arrows block continuity relative to the At-α derived paralog and the *Aethionema*-*Arabis* comparison.

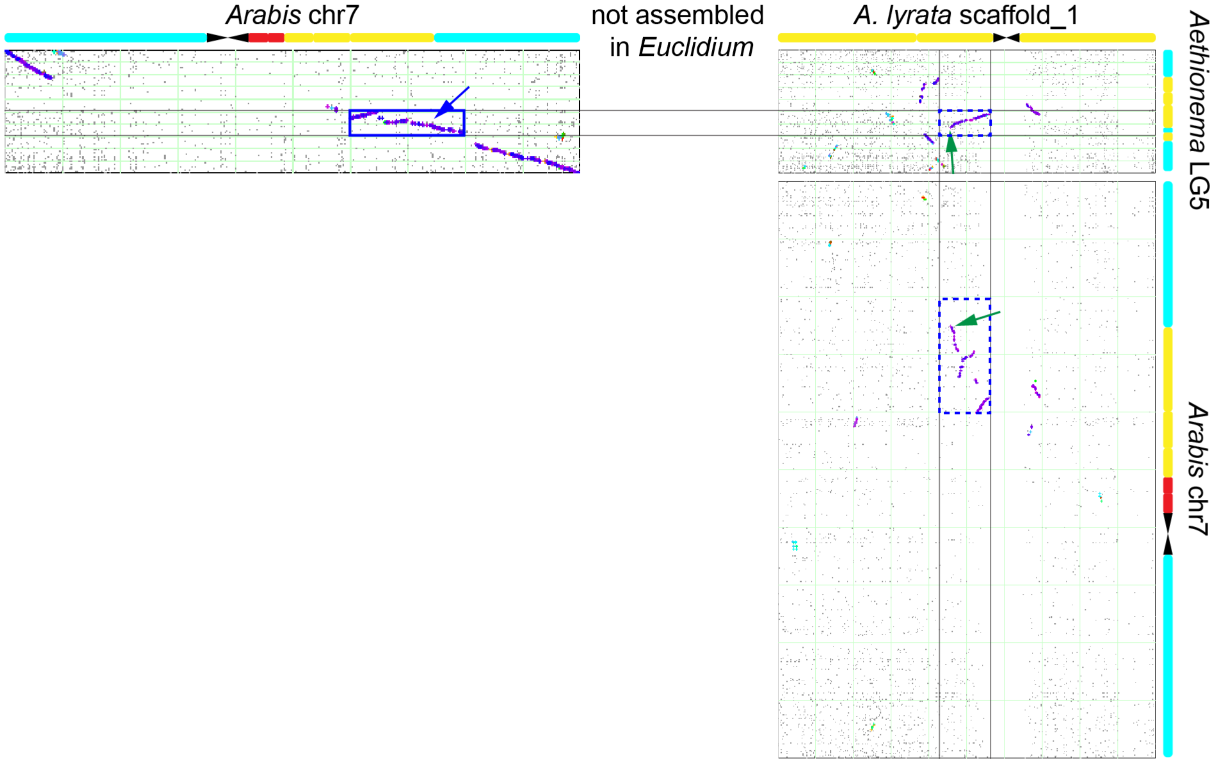

Supplementary Figure 6. Genomic block continuity from Fig. 3B. A detailed, chromosomal view of the break points shown in Fig. 3B in pairwise comparisons between *Aethionema*, *Arabis* and *Arabidopsis*. Orthologous regions in *Euclidium* were not assembled in one piece and no conclusion on block continuity in this species can be derived from the analyses; pairwise comparisons with *Euclidium* are therefore not displayed here. The genomic region continuous in the pairwise syntenic dotplot of *Aethionema* and *Arabis*, spanning the blocks U3-B5 in *Aethionema*, is highlighted in blue. Orthologs of this region in the other pairwise comparisons are highlighted with a blue dashed line. Green arrows indicate the break points, blue arrows block continuity relative to the At-α derived paralog and the *Aethionema*-*Arabis* comparison.

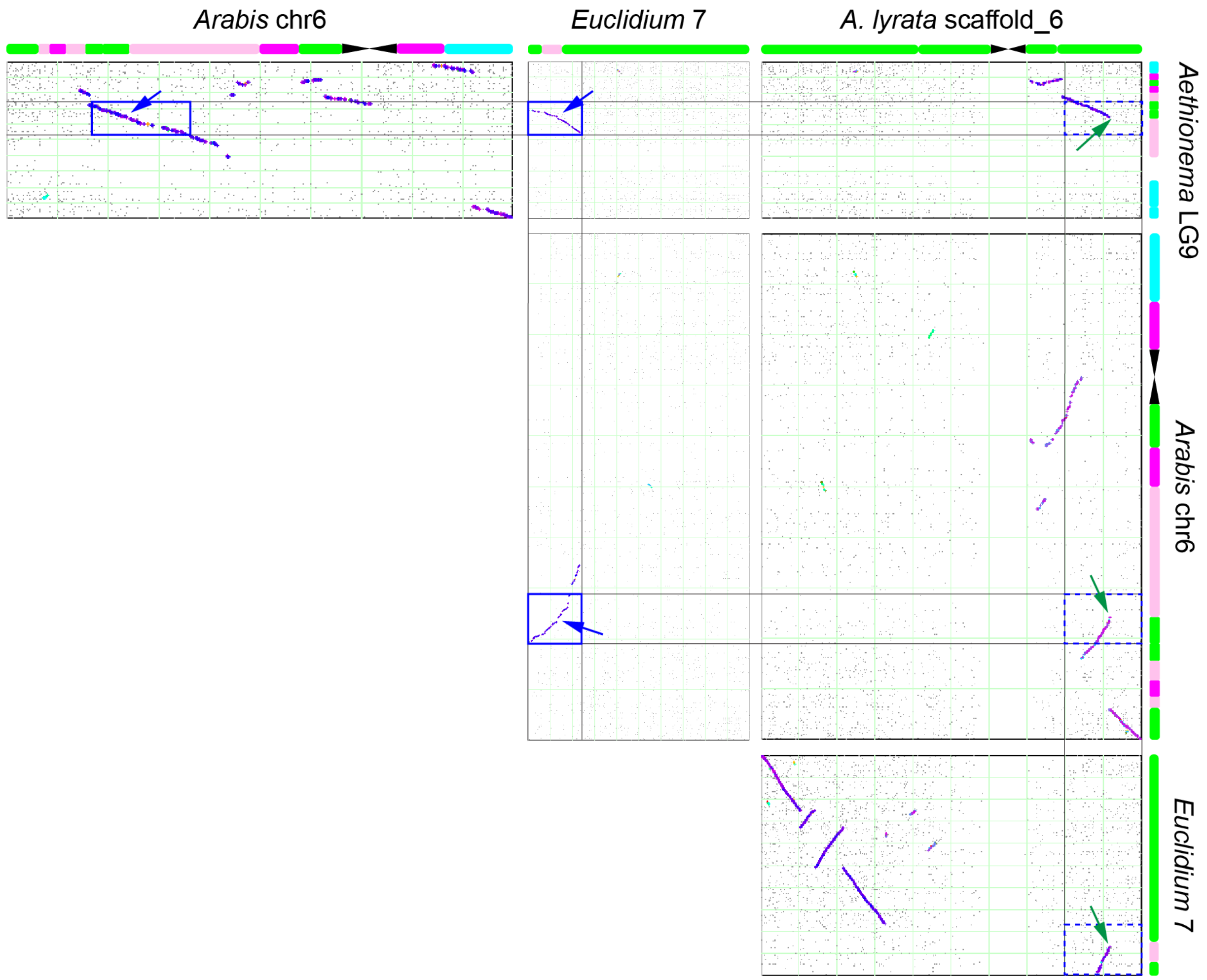

Supplementary Figure 7. Genomic block continuity from Fig. 3C. A detailed, chromosomal view of the break points shown in Fig. 3C in pairwise comparisons between *Aethionema*, *Arabis*, *Euclidium* and *Arabidopsis*. The genomic region continuous in the pairwise syntenic dotplot of *Aethionema* and *Arabis*, spanning the blocks V1-O2 in *Aethionema*, is highlighted in blue. Here, the same continuous syntenic block was detected in *Euclidium*. Non-continuous orthologs of this region in the other pairwise comparisons are highlighted with a blue dashed line. Green arrows indicate the break points, blue arrows block continuity relative to the At-α derived paralog and the *Aethionema*-*Arabis*-*Euclidium* comparisons.

1.3 References

Hu, T. T., Pattyn, P., Bakker, E. G., Cao, J., Cheng, J.-F., Clark, R. M., et al. (2011). The *Arabidopsis lyrata* genome sequence and the basis of rapid genome size change. *Nat Genet* 43, 476–481. doi:10.1038/ng.807.

Jiao, W.-B., Accinelli, G. G., Hartwig, B., Kiefer, C., Baker, D., Severing, E., et al. (2017). Improving and correcting the contiguity of long-read genome assemblies of three plant species using optical mapping and chromosome conformation capture data. *Genome Res.* 27, 778–786. doi:10.1101/gr.213652.116.

Mandáková, T., Hloušková, P., Koch, M. A., and Lysak, M. A. (2020). Genome evolution in Arabideae was marked by frequent centromere repositioning. *Plant Cell*, tpc.00557.2019. doi:10.1105/tpc.19.00557.

Nguyen, T.-P., Mühlich, C., Mohammadin, S., van den Bergh, E., Platts, A. E., Haas, F. B., et al. (2019). Genome improvement and genetic map construction for *Aethionema arabicum*, the first divergent branch in the Brassicaceae family. *G3: Genes|Genomes|Genetics* 9, 3521. doi:10.1534/g3.119.400657.

Willing, E.-M., Rawat, V., Mandáková, T., Maumus, F., James, G. V., Nordström, K. J. V., et al. (2015). Genome expansion of *Arabis alpina* linked with retrotransposition and reduced symmetric DNA methylation. *Nature Plants* 1, 14023. doi:10.1038/nplants.2014.23.
